# How to execute research projects in clinical practice in a large medium secure forensic psychiatric facility

**DOI:** 10.1192/j.eurpsy.2022.889

**Published:** 2022-09-01

**Authors:** A.M. Brandt-Christensn, E. Gruber, M. Christensen, H. Nerdrum, L.M. Pedersen, M. Sass, A. Fink-Jensen

**Affiliations:** 1 Copenhagen University Hospital, Psychiatric Centre Skt Hans, Department Of Forensic Psychiatry, Roskilde, Denmark; 2 Copenhagen University Hospital, Psychiatric Centre Copenhagen, Department Of Psychiatry Rigshospitalet, København Ø, Denmark; 3 Copenhagen University Hospital, Psychiatric Centre CopenhagenSkt Hans, Department Of Psychiatry Rigshospitalet, København Ø, Denmark

**Keywords:** executing clinical research, forensic psychiatry

## Abstract

**Introduction:**

While effective project planning is crucial for the success of a clinical research project, being able to execute the plan is even more important. In Denmark, approval for health research projects is applied for at regional or national committees on health research ethics, which have been reluctant to approve clinical research projects involving forensic psychiatric in-patients, due to the admission usually being pursuant to treatment sanctions. However, recently we received approval for a clinical research project exclusively targeted towards inpatients at a large medium secure forensic psychiatric facility in Denmark.

**Objectives:**

Describing the process of project execution from planning to submitting the manuscript which is inherently multi-faceted and inundated with stress factors. How to connect theory, knowledge, project with clinical practice, with clinical research?

**Methods:**

Qualitative data collecting while undertaking an exploratory, open-label, non-randomised weight reducing trial with a glucagon-like peptide-1 receptor agonist.

**Results:**

Challenges in finding, screening, motivating, recruiting, obtaining valid confirmed consent from potential study participants and other stakeholders, team communication, responsibilities and accountabilities within the team, Pareto Principle, scope creep, building project reports manually, real-time data gathering, unpredictable and other project deliverables will be presented

**Conclusions:**

Experiences of the hospital staff (psychiatrists, doctors and nurses) in execution process of the project investigation performed and made possible through participation of their forensic psychiatric in-patients.

**Disclosure:**

No significant relationships.

